# Folic Acid and Risk of Preterm Birth: A Meta-Analysis

**DOI:** 10.3389/fnins.2019.01284

**Published:** 2019-11-28

**Authors:** Bingbing Li, Xiaoli Zhang, Xirui Peng, Shan Zhang, Xiaoyang Wang, Changlian Zhu

**Affiliations:** ^1^Henan Key Laboratory of Child Brain Injury, Third Affiliated Hospital and Institute of Neuroscience of Zhengzhou University, Zhengzhou, China; ^2^Perinatal Center, Institute of Neuroscience and Physiology, University of Gothenburg, Sahlgrenska Academy, Gothenburg, Sweden; ^3^Center for Brain Repair and Rehabilitation, Institute of Neuroscience and Physiology, University of Gothenburg, Sahlgrenska Academy, Gothenburg, Sweden

**Keywords:** folate levels, folic acid supplementation, dietary folate intake, meta-analysis, preterm birth, preterm brain injury, sequelae of preterm birth

## Abstract

The results from epidemiologic studies linking blood folate concentrations, folic acid supplementation, or dietary folate to the risk of preterm birth are inconsistent. In this study, we aimed to summarize the available evidence on these associations. A systematic search of the PubMed/MEDLINE, Google Scholar, Web of Science, and Cochrane Library databases up to October 20, 2018 was performed and reference lists of retrieved articles were screened. Pooled odds ratios (ORs) and 95% confidence intervals (CIs) for the highest vs. the lowest levels of folate concentrations, folic acid supplementation, and dietary folate were calculated using random-effects models. Subgroup analyses and univariate meta-regression were performed to explore the sources of heterogeneity. Ten studies (six prospective cohort studies and four case-control studies) were included on folate concentrations, 13 cohort studies were included about folic acid supplementation, and 4 cohort studies were included regarding dietary folate intake. Higher maternal folate levels were associated with a 28% reduction in the risk of preterm birth (OR 0.72, 95% CI 0.56–0.93). Higher folic acid supplementation was associated with 10% lower risk of preterm birth (OR 0.90, 95% CI 0.85–0.95). In addition, a significant negative association was observed between dietary folate intake and the risk of preterm birth (OR 0.68, 95% CI 0.55–0.84), but no significant relation was seen between dietary folate and the risk of spontaneous preterm birth (OR 0.89, 95% CI 0.57–1.41). In the subgroup analysis, higher maternal folate levels in the third trimester were associated with a lower risk of preterm birth (OR 0.58, 95% CI 0.36–0.94). To initiate taking folic acid supplementation early before conception was adversely associated with preterm birth risk (OR 0.89, 95% CI 0.83–0.95). In conclusion, higher maternal folate levels and folic acid supplementation were significantly associated with a lower risk of preterm birth. The limited data currently available suggest that dietary folate is associated with a significantly decreased risk of preterm birth.

## Introduction

Preterm birth (PTB) and its associated complications, which include brain injury, retinopathy of prematurity, cerebral palsy, and developmental disabilities, are among the most serious global health issues. These complications directly affect the child's quality of life and are a huge burden both socially and economically (Goldenberg et al., 2008; Song et al., 2016; WHO, 2018). Thus, prevention of PTB is a global priority (Blencowe et al., 2013). Recent estimates of the incidence of PTB in most Europe countries range from 3.7 to 7.5% of live births (Poulsen et al., 2015). In the United States, the incidence is higher at about 9.62% (The Lancet, 2016). In Australia, the incidence was at 8.7% according to the latest annual report in 2015 (Hoh et al., 2019). In China, the incidence was at ~7% in 2016 (Chen et al., 2018). However, despite ongoing research, there has been no significant reduction in PTB rates. This might be a result of an inadequate understanding of the pathological processes contributing to PTB. PTB is considered a multifactorial syndrome, with almost 70% of PTBs resulting from spontaneous labor and/or rupture of membranes and the remainder from iatrogenic causes. Hence, it can be broadly categorized into spontaneous PTB (sPTB) and indicated PTB (Goldenberg et al., 2008). It has been recognized that there are numerous biological mechanisms that vary between individuals and that might lead to PTB (Frey and Klebanoff, 2016). Therefore, the identification of modifiable risk factors is of great importance for PTB management and prevention.

Maternal nutrition is an important determinant of the duration of pregnancy and fetal growth, and thereby influences pregnancy outcomes. Experimental data from animal studies suggest that maternal nutritional status such as folate status might play a role in PTB (Zhao et al., 2013; Scholl and Chen, 2015). Folate is an essential B vitamin that plays a role in DNA synthesis and cell division to support growth and fetal development (Lucock, 2000). During pregnancy, there is an increased demand for folate due to the rapid fetal growth. A previous study reported that pregnant women had a 5- to 10-fold higher folate requirement than non-pregnant women (Antony, 2007). Blood folate levels, including serum/plasma or red blood cell (RBC) folate, are considered reliable indicators of folate status (World Health Organization, 2015). Some recent epidemiological studies have indicated that low blood folate levels during pregnancy are associated with an increased risk of PTB (Bergen et al., 2012; Chen et al., 2014), while some other studies have shown no association between blood folate levels and PTB (Dunlop et al., 2012; Heeraman, 2016). In addition, a meta-analysis has not yet been conducted to summarize the epidemiological evidence on this association.

Folate cannot be synthesized by the body, and humans are entirely dependent on dietary sources or dietary supplements for their folate supply. There have been a large number of studies describing the association between folate intake and preterm birth (Vahratian et al., 2004; Lassi et al., 2013; Li et al., 2014; Mantovani et al., 2014; Martinussen et al., 2015; Zheng et al., 2016); however, their results are conflicting. Two meta-analyses from 2015 assessed the possible association between folic acid supplementation and the risk of PTB. One incorporated data from five randomized trials and reported no statistically significant effects (Saccone and Berghella, 2016), while the other identified nine observational studies and showed a decreased risk of PTB when initiating folic acid supplementation after conception (Zhang et al., 2017). Additional larger cohort studies have been published since then that might enhance the statistical power (Liu et al., 2016; Zheng et al., 2016), and thus an updated meta-analysis is needed.

In this study, we aimed to evaluate the available evidence on the associations between blood folate levels, dietary folate intake, and folic acid supplementation and the risk of PTB.

## Materials and Methods

### Literature Search

We conducted a literature search of PubMed (Medline), Google Scholar, Web of Science, and the Cochrane Library from their inception through October 2018. The search terms included “folic acid,” “vitamin B9,” “folate,” “folate status,” “folate levels,” “folate concentrations,” “serum folate,” “red blood cell folate,” “folic acid consumption,” “folic acid supplementation,” “folic acid intake,” “food folate,” or “dietary folate” combined with “preterm delivery,” “premature birth,” or “preterm birth.” We adhered to the Meta-analysis Of Observational Studies in Epidemiology (MOOSE) guidelines when undertaking this study (Stroup et al., 2000).

### Inclusion Criteria

Articles were included if (1) the study design was observational, (2) the population was healthy women who had the intention to become pregnant or who were pregnant, (3) the exposure of interest was folate levels or dietary folate intake or folic acid supplementation, (4) the outcome of interest was preterm birth, which was defined as delivery at <37 weeks gestation, (5) the association between folate levels or dietary folate intake or folic acid supplementation and risk of PTB was evaluated, and (6) adjusted risk estimates [relative risks (RRs), hazard risks (HRs), or odds ratios (ORs)] with their corresponding 95% confidence intervals (CIs) or standard errors were reported. Additionally, we excluded reviews, editorials, non-human studies, randomized clinical trials, and letters without sufficient data. We excluded randomized clinical trials without folic acid in a control group because of limited publications and ethical issues. When multiple reports based on the same study were published, only the most recent or complete report was used.

### Data Extraction

We extracted the following data from the included articles: the name of the first author, year of publication, country, study design, sample size, follow-up period, study period, assessment methods of folate levels, or dietary folate intake or folic acid supplementation, ascertainment of PTB, ORs and corresponding 95% CIs, and the confounding factors used for adjustment of the ORs. The ORs with more adjusted confounders were chosen when studies had different models for the calculation of estimated risks.

### Quality Assessment

Two reviewers independently performed the quality assessment using the Newcastle-Ottawa Scale (for cohort and case-control studies; Stang, 2010), which is a nine-point system including the selection process of studies (0–4 points), the comparability of studies (0–2 points), and the identification of the exposure and the outcomes of the study participants (0–3 points). The quality of articles was first evaluated according to the established questions, which were scored as 1 if the item was considered in the study or 0 if the item was not considered or if it was impossible to determine whether it was considered or not. We assigned scores of 0–3, 4–6, and 7–9 points for low, moderate, and high-quality studies, respectively (Supplementary Table 1).

### Statistical Analysis

Because most of included studies reported risk estimates as ORs, and because it was previously reported that ORs, HRs, and RRs provide similar estimates of risk when the incidence of outcome is very low (<10%) (Greenland, 1987), we chose ORs as the common effect size and combined HRs and RRs with ORs in the meta-analysis. The statistical analyses for the overall association between folate levels, folic acid supplementation/dietary folate intake, and PTB risk were based on comparisons of the highest category with the lowest. If the original studies did not provide corresponding data, the OR and its 95% CI were recalculated.

The ORs and corresponding 95% CIs were pooled using the DerSimonian and Laird random-effects model (DerSimonian and Laird, 1986), which considers both within-study and between-study variations. The summary measures were presented as forest plots where the sizes of the data markers (squares) correspond to the inverse of the variance of the natural logarithm of the OR from each study and the diamond indicates the pooled OR. Statistical heterogeneity among studies was quantified using the *I*^2^ statistic (Higgins et al., 2003).

To further evaluate the effects of heterogeneity, univariate meta-regression analyses were performed examining the effects of several key study characteristics. Stratified analyses by specimen gestational age, sample type, initiation time of folic acid supplementation, and dosage of folic acid were conducted to assess their impact on our estimates. Sensitivity analyses were employed to find potential origins of heterogeneity and to examine the influence of various exclusions on the combined OR. Funnel plots were used to assess small-study effects. Publication bias was assessed through the visual inspection of funnel plots and with tests of Begg rank correlation (Begg and Mazumdar, 1994). *P* < 0.05 was considered to be representative of a statistically significant publication bias. Forest plots were created to assess the overall association between folate levels, dietary folate, folic acid supplementation, and PTB.

All statistical analyses were performed with STATA version 12.0 software (Stata Corporation, College Station, TX, US). All reported probabilities (*P*-values) were two-sided, with *P* < 0.05 considered statistically significant, except for the Cochran's Q statistic in the heterogeneity test, in which the significance level was 0.10.

## Results

### Characteristics of the Studies

**Figure 1:**
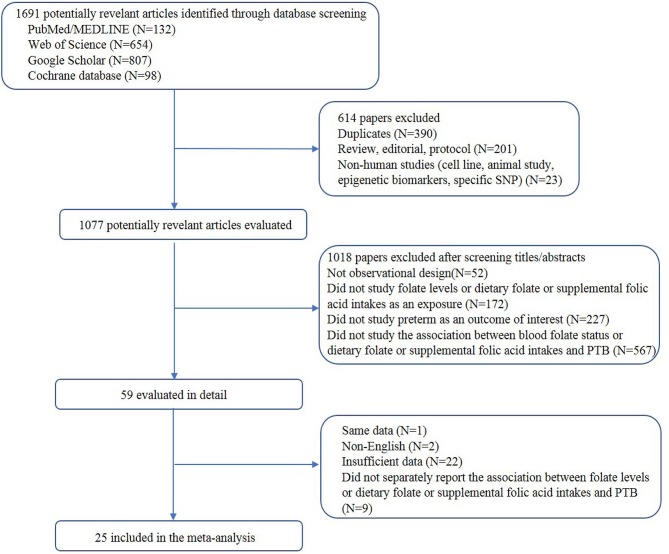
Flow chart for study selection (through October 20, 2018).

A total of 25 articles were included. The process of study selection is depicted in Figure 1. A total of 9 studies assessed the association between blood folate levels and the risk of PTB, 12 examined folic acid supplementation and the risk of PTB, and 2 studies assessed the association between dietary folate intake and the risk of PTB. Moreover, 1 study examined the association of folic acid supplementation and dietary folate and the risk of PTB, and 1 study assessed the association of blood and dietary folate with the risk of PTB.

### Blood Folate Levels and Risk of PTB

For the overall risk of PTB in relation to blood folate levels, we included 6 prospective cohort studies (Scholl et al., 1996; Siega-Riz et al., 2004; Bodnar et al., 2010; Bergen et al., 2012; Dunlop et al., 2012; Chen et al., 2014) and 4 case-control studies (Ronnenberg et al., 2002; Martí-Carvajal et al., 2004; Furness et al., 2012; Heeraman, 2016). A total of 7 studies measured folate levels in plasma/serum, and 3 studies measured folate levels in RBCs. Among these studies, 3 were sampled in the first trimester, 3 in the second trimester, and 3 in the third trimester. The main characteristics of these studies are summarized in Supplementary Tables 2, 3.

**Figure 2:**
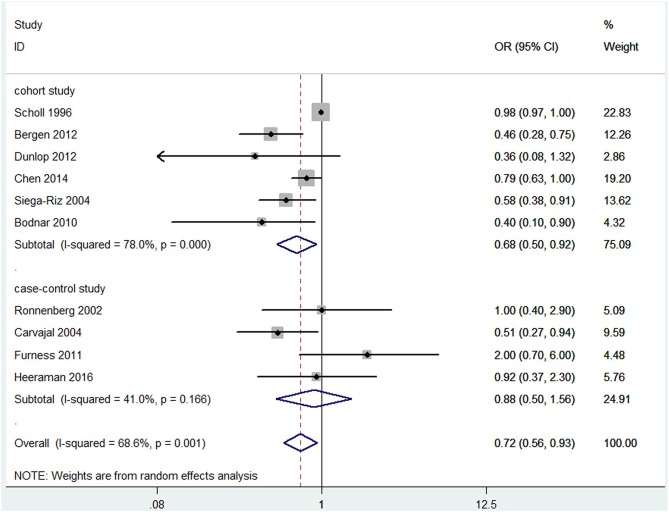
Forest plot of the meta-analysis of PTB risk in relation to blood folate levels, comparing the highest category with the lowest. The solid diamonds and horizontal lines indicate the study-specific ORs and 95% CIs. The size of the gray area reflects the study-specific statistical weight. The hollow diamonds represent the pooled ORs and 95% CIs of each subgroup and the overall population. The vertical solid line shows the OR of 1, and the vertical red dashed line represents the combined effect estimate.

Overall, a significant negative association between blood folate levels and PTB risk was observed. The pooled OR (95% CI) for PTB risk in individuals with the highest level of blood folate compared with the lowest level was 0.72 (95% CI, 0.56–0.93) with a moderate to high degree of statistical heterogeneity (*I*^2^ = 68.6%; Figure 2). For the 6 cohort studies, the negative association was consistent and the combined OR was 0.68 (95% CI, 0.50–0.92) with a high heterogeneity (*I*^2^ = 78%). For the 4 case-control studies, no significant association was found between blood folate levels and the risk of PTB (OR 0.88, 95% CI 0.50–1.56). Heterogeneity was observed between studies (*I*^2^ = 41%). To examine this heterogeneity, we conducted meta-regression analyses with type of design, gestational age of the specimen, geographical region, year of publication, and sample type as the independent variables. As shown in Table 1, no significant differences were found among these groups. No evidence of publication bias was observed when assessing the association between maternal folate levels and PTB (Begg's test, *P* = 0.592). A sensitivity analysis by omitting one study at a time did not dramatically influence the pooled ORs, suggesting that the combined OR was valid and credible.

**Table 1:** Blood folate levels and the risk of preterm birth analyzed by univariate meta-regression model.

**Covariate**	**Number of studies**	**β-coefficient**	***P*-value**
**Type of design**			
Cohort study, Case-control study	10	0.266	0.429
**Specimen gestational age**			
First trimester vs. Second trimester vs. Third trimester vs. preconception	10	−0.021	0.927
**Geographical region**			
Asia vs. US vs. Europe vs. Australia	10	0.084	0.597
**Sample size**			
≥1,000 vs. <1,000	10	0.444	0.083
**Year of publication**			
≥2010 vs. <2010	10	0.089	0.756
**Type of control**			
Hospital vs. Population	10	0.418	0.077
**Sample type**			
Serum/Plasma vs. Red blood cells	10	0.216	0.534

*The β-coefficient represents the change in log OR per unit increase in the relevant variable*.

**Figure 3:**
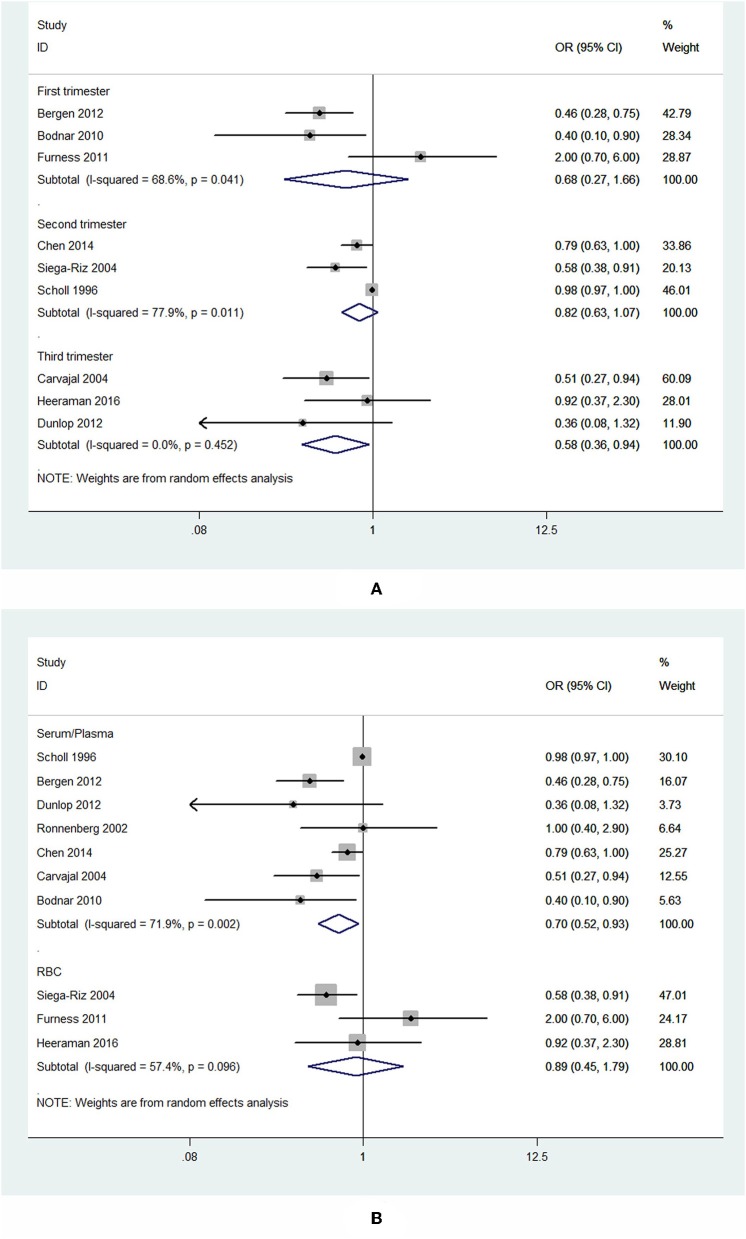
Forest plot of the meta-analysis of PTB risk in relation to blood folate stratified by gestational age of the specimen **(A)** and sample type **(B)**, comparing the highest category with the lowest. The diamonds and horizontal lines indicate the subgroup-specific ORs and 95% CIs. The size of the gray area reflects the study-specific statistical weight. The vertical solid line shows the OR of 1.

Subgroup analysis of specimen gestational age suggested no association between blood folate levels in the first (OR = 0.68, 95% CI 0.27–1.66, *P* = 0.393) and second trimester (OR = 0.82, 95% CI 0.63–1.07, *P* = 0.139) and the risk of PTB. However, the blood folate level in the third trimester was significantly inversely associated with the risk of PTB (OR = 0.58, 95% CI 0.36–0.94, *P* = 0.026; Figure 3A).

Subgroup analysis showed that higher plasma/serum folate was associated with 30% lower risk of PTB (OR = 0.70, 95% CI 0.52–0.93, *P* = 0.014), while RBC folate was not associated with the risk of PTB (OR = 0.89, 95% CI 0.45–1.79, *P* = 0.75; Figure 3B).

### Folic Acid Supplementation and Risk of PTB

Of the 13 cohort studies that assessed folic acid supplementation and the overall risk of PTB (Scholl et al., 1997; Vahratian et al., 2004; Catov et al., 2007, 2011; Timmermans et al., 2009; Alwan et al., 2010; Czeizel et al., 2010; Papadopoulou et al., 2013; Li et al., 2014; Martinussen et al., 2015; Liu et al., 2016; Zheng et al., 2016; Baron et al., 2017), 6 studies reported two separate outcomes stratified by initiation time of supplementation (preconception and postconception). In this case, each of studies could be considered as two independent reports. Thus, there were 19 independent reports included in this meta-analysis. Among these studies, 4 used folic acid alone and the rest used folic acid containing multivitamins. A total of 9 stated the exact content of folic acid supplements, and 7 out of the 9 studies used folic acid at 400 μg daily (as suggested by WHO), and 2 used high doses of folic acid of more than 1,000 μg daily. Overall, the lowest category (reference category) observed in the included studies ranged from 0 to 200 μg daily, and the highest category ranged from any folic acid/folic acid-containing supplements consumption to ≥1,000 μg daily. Because we used the categories reported by the studies, these categories were not mutually exclusive. None of the studies described the mutual effects associated with multivitamins. The main characteristics of these studies are summarized in Supplementary Table 4.

**Figure 4:**
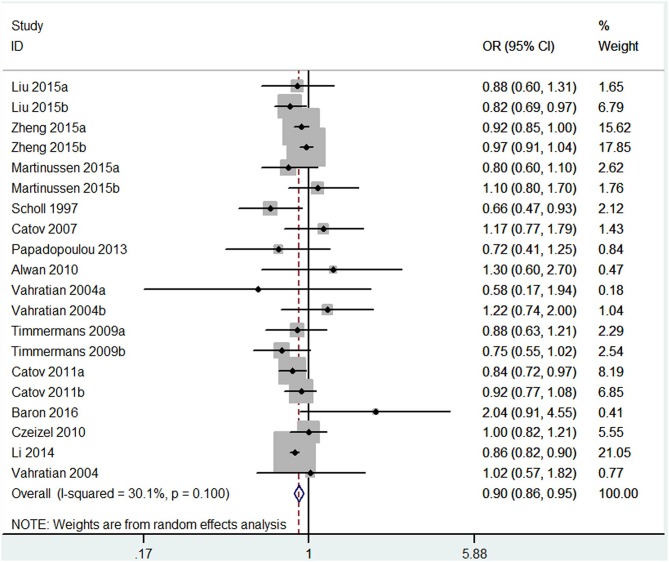
Forest plot of the meta-analysis of PTB risk in relation to folic acid supplementation, comparing the highest category with the lowest. The diamonds and horizontal lines indicate the corresponding ORs and 95% CIs. The size of the gray area reflects the study-specific statistical weight. The vertical solid line shows the OR of 1, and the vertical red dashed line represents the combined effect estimate. The suffix “a” or “b” after the studies indicates two separate outcomes stratified by the initiation time of supplementation (preconception and post-conception) in the same study.

**Figure 5:**
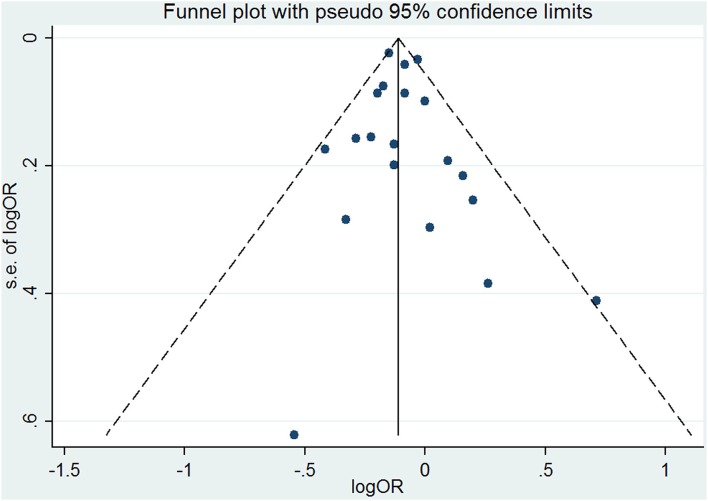
Funnel plot for studies of folic acid supplementation in relation to PTB risk. The vertical solid line represents the summary effect estimates, and the dotted lines are pseudo 95% CIs.

The results combining the ORs comparing the highest and lowest category of folic acid supplementation for the risk of PTB are shown in Figure 4. An inverse association was found (OR = 0.90, 95% CI 0.86–0.95), and low to moderate heterogeneity across the studies was found (*I*^2^ = 30.1%). To examine this heterogeneity, we conducted meta-regression analyses with initiation time of supplementation, geographical region, source of cohorts, and ascertainment of PTB, sample size, year of publication and dosage of folic acid intake. As shown in Table 2, no significant differences were observed among these subgroups. Visual inspection of the funnel plot showed little asymmetry for studies on folic acid supplementation and PTB risk (Figure 5). No evidence of publication bias was found across the included studies (Begg's test, *P* = 0.284), and sensitivity analyses using a fixed-effect model or omitting one study at a time did not substantially alter the pooled results.

**Table 2:** Folic acid supplementation and the risk of preterm birth analyzed by univariate meta-regression model.

**Covariate**	**Number of studies**	**β-coefficient**	***P*-value**
**Time of FA intake**			
Preconception vs. Postconception vs. Periconception	19	0.014	0.829
**Geographical region**			
Asia vs. US vs. Europe	18^*^	−0.0008	0.98
**Sample size**			
≥1,000 vs. <1,000	19	−0.012	0.877
**Year of publication**			
≥2010 vs. <2010	19	−0.054	0.565
**Source of Cohort**			
Hospital vs. Population	19	0.012	0.875
**Definition of GA**			
LMP vs. Ultrasound vs. Both	19	−0.009	0.977
**Dose of intake**			
Moderate vs. High	19	0.067	0.571

* *One study did not provide data for geographical region. The β-coefficient represents the change in log OR per unit increase in the relevant variable. FA, folic acid; LMP, the last normal menstrual period; GA, gestational age*.

**Figure 6:**
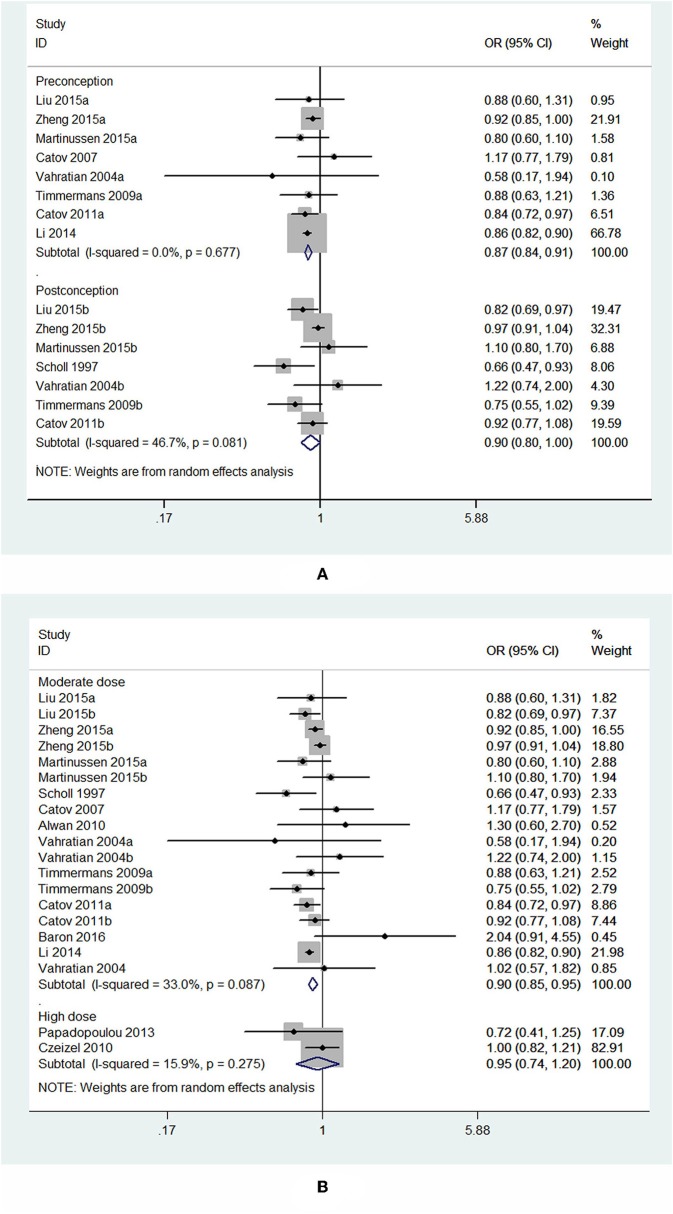
Forest plot of the meta-analysis of PTB risk in relation to folic acid supplementation stratified by initiation time **(A)** and dose of folic acid intake **(B)**, comparing the highest category with the lowest. The diamonds and horizontal lines indicate the subgroup-specific ORs and 95%CIs. The size of the gray area reflects the study-specific statistical weight. The vertical solid line shows the OR of 1. The suffix “a” or “b” after the studies indicates two separate outcomes stratified by initiation time of supplementation (preconception and post-conception) in the same study.

Subgroup analysis of initiation time of folic acid supplementation showed that initiating folic acid supplements before conception was associated with a significant decreased risk of PTB (OR = 0.87, 95% CI: 0.84–0.91, *P* < 0.001), while starting folic acid supplementation at post-conception was associated with a marginal decreased risk of PTB (OR = 0.90, 95% CI: 0.80–1.00, *P* = 0.049; Figure 6A).

In the analysis stratified by dose of folic acid intake, a statistically significant protective effect was noted between folic acid supplementation at a daily dosage of <1,000 μg and PTB risk (OR = 0.90, 95% CI: 0.85–0.95, *P* < 0.001). However, taking folic acid supplementation at a daily dosage of more than 1,000 μg was not significantly associated with the risk of PTB (OR = 0.95, 95% CI: 0.74–1.20, *P* = 0.65; Figure 6B).

### Dietary Folate Intake and Risk of PTB

Of the 4 cohort studies that assessed dietary folate intake and risk of PTB (Siega-Riz et al., 2004; Shaw et al., 2011; Sengpiel et al., 2014; Liu et al., 2016), 1 study reported two separate outcomes stratified by the initiation time of supplementation (preconception and post-conception). Thus, there were 5 independent reports, including 95,448 participants, in our meta-analysis. Out of these 5 studies, 2 studies reported the outcome of sPTB in addition to overall PTB, 2 studies just reported sPTB, and 1 study just reported overall PTB. In total, 3 studies reported the overall risk of PTB in relation to dietary folate intake, and 4 studies reported sPTB. The main characteristics of these studies are summarized in Supplementary Tables 5, 6.

**Figure 7:**
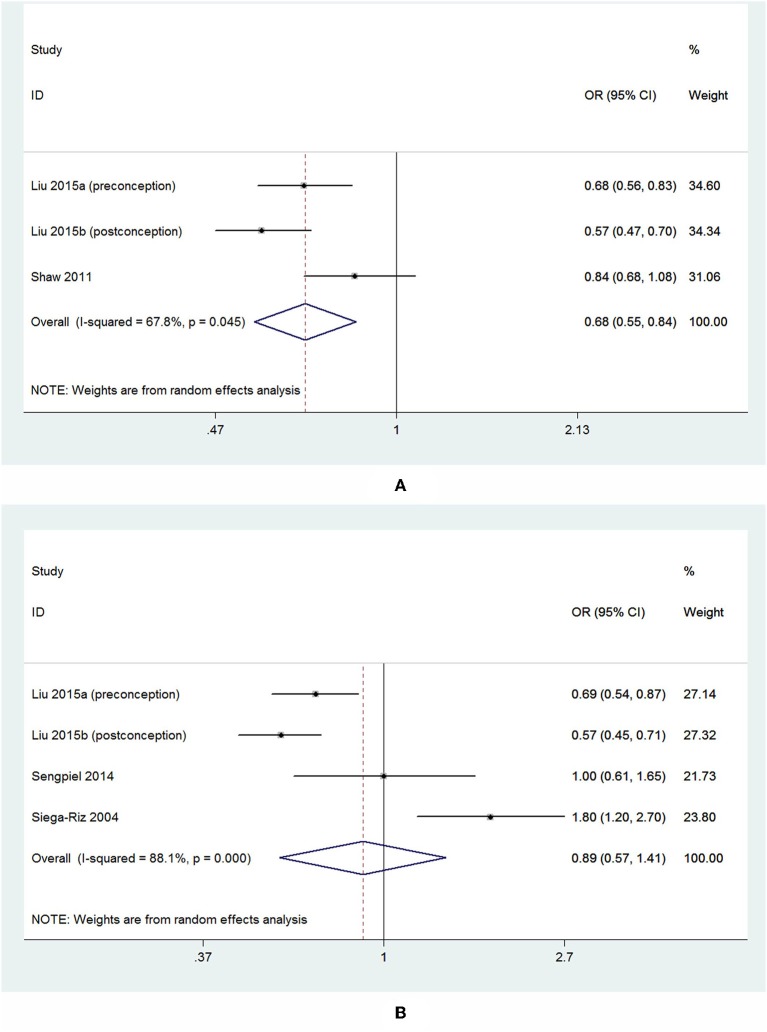
Forest plot of the meta-analysis of PTB risk **(A)** and sPTB risk **(B)** in relation to dietary folate, comparing the highest category with the lowest. The diamonds and horizontal lines indicate the corresponding ORs and 95% CIs. The size of the gray area reflects the study-specific statistical weight. The vertical solid line shows the OR of 1, and the vertical red dashed line represents the combined effect estimate.

For the 3 studies about overall PTB, there was a significant inverse association observed between dietary folate intake and the overall risk of PTB, and the pooled OR and 95% CI for PTB when comparing the highest with the lowest levels of dietary folate intake was 0.68 (95% CI 0.55–0.84) with a moderate heterogeneity (*I*^2^ = 67.8%; Figure 7A). For the 4 studies about sPTB, a non-significant association was observed. The summary OR and 95% CI was 0.89 (95% CI 0.57–1.41) with a high heterogeneity (*I*^2^ = 88.1%; Figure 7B). We did not perform subgroup analysis or sensitivity analysis due to the small number of studies.

## Discussion

### Principal Findings of This Study

The current meta-analysis is the first time to assess the association of PTB risk with blood folate levels and dietary folate intake. We found that blood folate levels, folic acid supplementation, and dietary folate intake were negatively associated with the overall risk of PTB. Furthermore, we found that dietary folate intake was not significantly associated with the risk of sPTB.

Compared to the previous meta-analysis on folic acid supplementation and risk of PTB, which included 9 studies, this updated meta-analysis included 13 studies and increased the sample size from 306,695 to 562,068 participants, which increased the statistical power of our analysis. Moreover, the included studies used modern methods of multivariate adjustment rather than raw data.

Evidence from biological studies supports a role of folate in PTB. First, folate contributes to oocyte maturation and early placentation (Jongbloet et al., 2008; Koukoura et al., 2012). Folate deficiency may lead to poor placentation and influence the development of and maintenance of uteroplacental circulation (Baker et al., 2017), which subsequently triggers poor pregnancy outcomes including PTB (Engel et al., 2006; Bailey, 2009). It has been observed that folate transporters, which transfer folate from maternal circulation to the fetus, are present at lower concentrations in preterm placentas compared to term placentas (Castaño et al., 2017). Second, folate is also a cofactor in the metabolism of homocysteine, which might be a contributing factor for placental vascular disease (Van der Molen et al., 2000), and epidemiological studies have shown that elevated homocysteine concentrations are associated with PTB (Bergen et al., 2012; Chen et al., 2014). Third, folate status during pregnancy might play an anti-inflammatory role. Many cases of PTB are associated with an abnormal inflammatory response, which is often caused by intrauterine infection and inflammation (Goldenberg et al., 2000). In a mouse model of lipopolysaccharide-induced PTB, folate reduced the levels of circulating biomarkers of inflammation, including interleukin (IL)-6 and keratinocyte-derived cytokine in the amniotic fluid of mice (Zhao et al., 2013).

### Blood Folate Levels and Risk of PTB

We found an inverse association between blood folate levels in the third trimester and the risk of PTB, while no significant association was observed in the first and second trimester. This might be explained by the following mechanisms. Placental dysfunction is one of the risk factors for PTB (Romero et al., 2014), and it was observed that maternal blood folate levels decreased from the fifth month of pregnancy and plasma homocysteine concentrations increased in later pregnancy (Wang et al., 2016). Thus, we hypothesized that inadequate third trimester maternal folate levels impact fetal development by adversely affecting placental function during the period of maximal fetal development. Previous studies have reported that the persistence of placental dysfunction from the 24th week of pregnancy is associated with increased risks of adverse pregnancy outcomes (López-Quesada et al., 2004; Gaillard et al., 2013). On the other hand, folate might also be indirectly involved in placental development through its role in the homocysteine cycle. Folate deficiency may disrupt the function of the enzymes in homocysteine metabolism and lead to an increase in homocysteine levels. It was reported that elevated homocysteine concentration is also associated with oxidative stress, arteriolar constriction, endothelial damage, and placental thrombosis, all of which increase the risk of pregnancy complications (Maged et al., 2017). Moreover, folate is an important methyl-group vitamin, and maternal plasma folate levels are associated with offspring DNA methylation, which is in turn related to fetal development. It was reported that maternal folate levels in late pregnancy are more important than folate levels in early pregnancy for overall fetal growth (Sulaiman et al., 2017). Thus, an inverse association was evident between maternal folate levels in the third trimester and the risk of PTB, and maternal folate levels in the third trimester might be an indirect predictor of PTB.

In general, the RBC folate concentration is generally considered to reflect folate status during the preceding 3–4 months, and plasma or serum folate is a short-term measure reflecting fluctuation of dietary/supplement intakes over the past month (He et al., 2016). Theoretically, the two different folate indicators are likely to be one source of heterogeneity. Although no evidence was found that the sample type might have affected results, our meta-analysis suggested that there was no significant association between RBC folate and PTB risk, but a negative association was identified between plasma/serum folate and PTB risk. Additionally, the higher *I*^2^ for the plasma/serum subgroup compared to RBCs suggests that different methods for measuring blood folate might be potential reasons for the heterogeneity, and the difference between serum/plasma and RBCs might had affected the *I*^2^ results to some degree.

### Folic Acid Supplementation and Risk of PTB

In accordance with a previous meta-analysis of observational studies, we found that folic acid supplementation reduced the risk for PTB. Moreover, we found that starting folic acid supplementation before conception was more effective in reducing the risk of PTB compared with post-conception. A population-based mega-cohort study came to the same conclusion as us (Nijhout et al., 2004). This seems biologically plausible given that folate has a half-life of 100 days (Tamura and Picciano, 2006). It has been well-established that folate concentrations in the circulation decline as pregnancy advances (Pickell et al., 2011), and it has been shown that starting folic acid supplementation before conception significantly increases maternal RBC folate concentrations and prevents the decline in serum folate concentration after pregnancy, and this might be beneficial to fetal growth (Bailey, 2009).

There have been concerns that high folic acid intake might be linked to abnormal embryonic development and long-term negative health outcomes in the offspring of mice (Dwarkanath et al., 2013) and humans (Shaw et al., 2004), therefore, it is necessary to evaluate the association between high folic acid intake and the risk of PTB. However, a meta-analysis of randomized controlled trials found that higher folic acid supplementation had no significant reduction of PTB risk (Saccone and Berghella, 2016). Another meta-analysis found that high folic acid reduced the risk of PTB (Zhang et al., 2017). In this meta-analysis, we found that folic acid supplementation was effective in reducing the risk of PTB only if the daily dose was <1,000 μg (moderate dose group), and folic acid supplementation ≥1,000 μg per day (high dose group) did not influence the risk of PTB. The cutoff of 1,000 μg of folic acid supplementation was chosen because most prenatal vitamins contain <1,000 μg folic acid (Bailey, 2009).

### Dietary Folate Intake and Risk of PTB

In this meta-analysis, the included 3 studies were unable to differentiate between spontaneous preterm birth and iatrogenic preterm birth and thus the two were equated. We found that dietary folate intake showed a strong inverse association with the overall risk of PTB, which was consistent with a previous a cross-sectional study (Deniz et al., 2018). As for the 4 studies specifically examining sPTB, dietary folate intake showed no significant reduction in the risk of sPTB. These results should be interpreted with caution due to the limited data, however, and future studies are required to address these issues.

### Limitations of the Study

There were several limitations in the present meta-analysis. First, we did not include RCTs in the current meta-analysis. Because of ethical issues, few RCTs have been conducted that have studied the association between folic acid supplementation and PTB compared to placebo or to no supplementation. Second, there were not enough studies to explore the dose-response trend of blood folate levels and folic acid supplementation in relation to PTB risk. Third, only a relatively small number of studies on the association between dietary folate intake and PTB risk have been published, so conclusions should be drawn with caution. Moreover, we defined folic acid supplementation as folic acid alone or folic acid-containing vitamins, and this might have led to the introduction of clinical heterogeneity. We were also unable to assess the mutual effect of multivitamins because of insufficient information. In addition, major risk factors for PTB, including socioeconomic status, lifestyle factors, and adverse health behaviors were difficult to control for in the included studies.

## Conclusions

In summary, the results of the present meta-analysis suggest that higher folate levels and folic acid supplementation are significantly associated with a lower overall risk of overall PTB. Dietary folate intake seemed to be significantly associated with a decreased risk of overall PTB and was not associated with risk of sPTB. However, this should be interpreted with caution because of the small number of studies. Subgroup analyses indicated that higher maternal folate levels in late pregnancy are associated with lower PTB risk and that initiating folic acid supplementation early before conception has a significant protective effect against PTB.

Therefore, considering the increasing numbers of preterm infants and recent reports on the neuroprotective effect of folate intake during pregnancy (Julvez et al., 2009), pregnant women should reinforce and start folate intake early before conception in order to reduce the risk of PTB and subsequent risks for long-lasting neurodevelopmental impairments. Additionally, moderately decreased folate levels in late pregnancy might increase the risk of PTB, and this will help with clinical risk stratification and patient counseling.

## Author Contributions

BL conceptualized and designed the study, collected and organized the data, and drafted the initial manuscript. XZ collected and organized the data, reviewed the included articles, and conducted the analyses. XP and SZ collected and organized the data and reviewed the included articles. XW conceptualized and designed the study and critically reviewed and revised the manuscript. CZ conceptualized and designed the study, coordinated and supervised data collection, and critically reviewed and revised the manuscript. All authors read and approved the final manuscript.

### Conflict of Interest

The authors declare that the research was conducted in the absence of any commercial or financial relationships that could be construed as a potential conflict of interest.
