# Supplementary material for: Folic Acid and Risk of Preterm Birth: A Meta-Analysis
Source: Front Neurosci. 2019 Nov 28;13:1284. doi: 10.3389/fnins.2019.01284 (PMC6892975; doi:10.3389/fnins.2019.01284)
Supplement: Supplementary file 1 [file Table_1.DOCX]

**Supplementary Table 1.** Assessment of quality of included studies for meta-analysis.

| **Studies** | **Quality assessment criteria^§^** | | | |
| --- | --- | --- | --- | --- |
|  | **Selection** | **Comparability** | **Exposure/Outcome** | **Overall quality** |
| Scholl 1996 | ** | ** | ** | 6 |
| Scholl 1997 | ** | ** | ** | 6 |
| Martinussen | *** | ** | ** | 7 |
| Liu | *** | ** | *** | 8 |
| Sengpiel | **** | ** | ** | 8 |
| Vahratian | **** | ** | ** | 8 |
| Timmermans | *** | ** | ** | 7 |
| Li | *** | ** | ** | 7 |
| Shaw | ** | ** | *** | 7 |
| Zheng | *** | ** | *** | 8 |
| Baron | **** | ** | ** | 8 |
| Catov 2007 | *** | ** | ** | 7 |
| Catov 2011 | *** | ** | *** | 8 |
| Czeizel | *** | ** | ** | 7 |
| Heeraman | *** | ** | *** | 8 |
| Papadopoulou | *** | ** | *** | 8 |
| Alwan | *** | ** | ** | 7 |
| Siega-Riz | *** | ** | ** | 7 |
| Bergen | **** | ** | *** | 9 |
| Dunlop | *** | ** | ** | 7 |
| Ronnenberg | *** | ** | ** | 7 |
| Chen | *** | ** | *** | 8 |
| Carvajal | *** | ** | *** | 8 |
| Furness | *** | ** | ** | 7 |
| Bodnar | *** | ** | ** | 7 |

^§^The study quality was assessed according to the Newcastle Ottawa Quality assessment scale for observational studies. ** 2 points. *** 3 points. **** 4 points.
