# Supplementary material for: Folic Acid and Risk of Preterm Birth: A Meta-Analysis
Source: Front Neurosci. 2019 Nov 28;13:1284. doi: 10.3389/fnins.2019.01284 (PMC6892975; doi:10.3389/fnins.2019.01284)
Supplement: Supplementary file 2 [file Table_2.DOCX]

**Supplementary Table 2.** Characteristics of the cohort studies (n = 6) regarding the associations between blood folate levels and the risk of preterm birth.

| **Reference** | **Country** | **Total included** | **Study years** | **Exposure Analysis method** | **Sample type** | **Specimen gestational age (week)** | **Folate levels (ng/ml)** | **OR (95% CI)** | **Adjustment factors** |
| --- | --- | --- | --- | --- | --- | --- | --- | --- | --- |
| Scholl 1996 | US | 832 | N/A | RIA | serum | 28 | N/A | 0.98(0.97–1.00) | gestation at entry, ethnicity, prior history of preterm delivery or LBW, maternal age, and parity |
| Bergen 2012 | The Netherlands | 5,805 | 2002.4–2006.1 | IECLA | plasma | 13.2 | highest (≥ 11.4) versus lowest (< 4.1) | 0.46 (0.28–0.75) ^*^ | gestational age at blood sampling, offspring sex, maternal age at intake, parity, educational level, geographical origin, comorbidity, maternal height, maternal weight at intake, smoking, and alcohol and caffeine use |
| Dunlop 2012 | US | 1,547 | 2003–2006 | CBCL-RIA | serum | at labor | ≥ 5 versus <5 | 0.36 (0.08–1.32) ^*^ | age, BMI, health care payer, marital status |
| Chen 2014 | Singapore | 999 | 2009.6–2010.9 | ECLI | plasma | 26th–28th | highest (≥19.6) versus lowest (<5.5) | 0.79 (0.63-1.00) | infant sex, ethnicity, maternal age, gravidity, maternal height, prepregnancy BMI, weight gain up until 26 weeks, educational level, and gestational diabetes mellitus |
| Siega-Riz 2004 | US | 3,164 | 1995–2000 | RA | RBC | 24-29 | highest (≥1440) versus lowest (<325) | 0.58 (0.38–0.91) ^*^ | prenatal supplement use and batch number and batch interaction terms |
| Bodnar 2010 | US | 313 | 2003–2007 | IMS | serum | 9.5(mean) | highest (≥38.9) versus lowest (<16.8) | 0.4 (0.10–0.90) | race-ethnicity, education, smoking, and obesity |

Abbreviations: **OR**, odds ratio; **CI**, confidence interval; **RBC**, red blood cell; **LBW**, low birth weight; **BMI**, body mass index; **RIA**, radioimmunoassay; **IECLA**, Immunoelectrochemoluminescence assay; **CBCL-RIA,** competitive-binding chemiluminescent radioimmunoassay; **ECLI**, electrochemiluminescence immunoassay; **RA**, radioassay kit; **IMS**, ionization mass spectrometry.

^*^ OR that used the lowest category of blood folate as a reference were recalculated using the lowest category as a reference to be included in the meta-analysis.
