# Supplementary material for: Folic Acid and Risk of Preterm Birth: A Meta-Analysis
Source: Front Neurosci. 2019 Nov 28;13:1284. doi: 10.3389/fnins.2019.01284 (PMC6892975; doi:10.3389/fnins.2019.01284)
Supplement: Supplementary file 3 [file Table_3.DOCX]

**Supplementary Table 3.** Characteristics of the case-control studies (n = 4) regarding the associations between blood folate levels and the risk of preterm birth.

| **Reference** | **Country** | **Total included** | **Study years** | **Exposure Analysis method** | **Sample type** | **Specimen gestational age (week)** | **Folate levels (ng/ml)** | **OR (95% CI)** | **Adjustment factors** |
| --- | --- | --- | --- | --- | --- | --- | --- | --- | --- |
| Ronnenberg 2002 | China | 1,280 | 1996.8–1998.12 | RIA | plasma | preconception | ≥3 versus <3 | 1.00 (0.40–2.90) | maternal age, BMI, hemoglobin concentration, and analytic batch |
| Carvajal 2004 | Spain | 543 | 1996.5–1996.12 | RIA | serum | at labor | ≥3 versus <3 | 0.51 (0.27–0.94) | prior preterm labor, prenatal care visits, prior abortion, prior fetal death, placental abruption, and premature rupture oval membranes |
| Furness 2011 | Australia | 400 | 2003–2006 | RIA | RBC | 10–12 | highest (≥400) versus lowest (<200) | 2.00 (0.70–6.00) | BMI, smoking, and maternal age |
| Heeraman 2016 | US | 227 | 2011-2013 | N/A | RBC | at labor | ≥952 versus <952 | 0.92 (0.37-2.30) ^*^ | smoking, history of preterm births |

Abbreviations: **OR**, odds ratio; **CI**, confidence interval; **RBC**, red blood cell; **BMI**, body mass index; **RIA**, radioimmunoassay.

^*^ OR that used the lowest category of blood folate as a reference were recalculated using the lowest category as a reference to be included in the meta-analysis.
