# Supplementary material for: Folic Acid and Risk of Preterm Birth: A Meta-Analysis
Source: Front Neurosci. 2019 Nov 28;13:1284. doi: 10.3389/fnins.2019.01284 (PMC6892975; doi:10.3389/fnins.2019.01284)
Supplement: Supplementary file 4 [file Table_4.DOCX]

**Supplementary Table 4.** Characteristics of the cohort studies (n = 19) regarding the associations between folic acid supplementation and the risk of preterm birth.

| **Reference** | **Country** | **Total included** | **Study years** | **Exposure analysis method** | **Folic acid intake (µg/day)** | **OR (95% CI)** | **Adjustment factors** |
| --- | --- | --- | --- | --- | --- | --- | --- |
| Li 2014 | China | 207,936 | 1993.10–1995.9 | FFQ | Never  Preconception/400 | 1.00 (reference)  0.86 (0.82–0.90) | maternal age (continuous), BMI (continuous), education, occupation, ethnicity and parity |
| Liu 2015a | China | 10,179 | 2010–2012 | SFFQ | Never  Preconception/400 | 1.00 (reference)  0.88 (0.60–1.31) | maternal age, education level, smoking, parity, preeclampsia, maternal diabetes, pre-pregnancy BMI, family monthly income per capita, maternal employment during pregnancy, history of preterm, and dietary folate intake |
| Liu 2015b | China | 10,179 | 2010–2012 | SFFQ | Never  Postconception/400 | 1.00 (reference)  0.82 (0.69–0.97) |  |
| Zheng 2015a | China | 231,179 | 1999–2012 | interview | Never  Preconception/400 | 1.00 (reference)  0.92 (0.85–1.00) | maternal age (continuous), BMI (continuous), menarcheal age (continuous), parity status, educational status (<high school, high school, >high school), occupational status (farm work, routine job, temporary job, unemployed), maternal residence (rural v. urban), child’s sex and recruiting year (<2010 vs. ≥2010). |
| Zheng2015b | China | 231,179 | 1999–2012 | interview | Never  Postconception/400 | 1.00 (reference)  0.97 (0.91–1.04) |  |
| Martinussen 2015a | US | 3,647 | 1996.9–2000.1 | FFQ | No use (<200)  Preconception (>200) | 1.00 (reference)  0.90 (0.6–1.1) | study, maternal age, maternal ethnicity, maternal education, maternal marital status, parity, maternal smoking in pregnancy and any miscarriages or stillbirths in previous pregnancies |
| Martinussen 2015b | US | 3,647 | 1996.9–2000.1 | FFQ | No use (<200)  Postconception (>200) | 1.00 (reference)  1.30 (0.9–1.8) |  |
| Scholl 1997 | US | 1,430 | 1985–1995 | interview | Never  Postconception (<1000) | 1.00 (reference)  0.53 (0.35–0.81) | age, parity, ethnicity, clinic payment status, inadequate weight gain for gestation, cigarettes/day, preconception body mass index, prior preterm delivery, 1st trimester bleeding and nausea, caloric intake, and preconception vitamin use. |
| Catov 2007 | US | 1,823 | 1997-2001 | interview | Never  Preconception (<1000) | 1.00 (reference)  1.17 (0.77–1.79) | race, age, education, gestational age at interview, household density, marital status, body mass index, smoking, moderate physical activity, greater than 30 hours of television watching per week, parity, preeclampsia, transient hypertension, and family history of preeclampsia |
| Papadopoulou 2013 | Spain | 1,279 | 2007.2 | FFQ | Never  Periconception (≥5000) | 1.00 (reference)  0.72 (0.41–1.25) | maternal age, education, Greek origin, pre-pregnancy BMI (kg/m^2^), smoking status, parity, iron intake from supplements |
| Alwan 2010  Vahratian 2004a  Vahratian 2004b  Vahratian 2004 | UK  US  US  US | 1,274  2010  2010  2010 | 2003-2006  1995.8-2000.6  1995.8-2000.6  1995.8-2000.6 | FFQ  FFQ  FFQ  FFQ | Never  Periconception (<1000)  Never  Preconception (<1000)  Never  Postconception (<1000)  Never  Periconception (<1000) | 1.00 (reference)  1.30 (0.6, 2.7)  1.00 (reference)  0.58 (0.17–1.94)  1.00 (reference)  1.22 (0.74–2.0)    1.00 (reference)  1.02 (0.57–1.82) | salivary cotinine levels, self-reported alcohol intake, maternal age, maternal vegetarian diet, ethnicity, baby’s sex, parity, educational attainment, past history of miscarriage, and long-term chronic illness  maternal health during pregnancy, vomiting during pregnancy, estimated energy intake,  daily iron per 1,000 kcal, daily folate per 1,000 kcal, parity, maternal race, and marital status |
| Timmermans 2009a | The Netherlands | 6,353 | 2002.4–2006.1 | FFQ | Never  Preconception/400 | 1.00 (reference)  0.88 (0.63–1.21) | gestational age at birth (not prematurity), maternal age, height, weight, parity, ethnicity, fetal gender, educational level, and smoking |
| Timmermans 2009b | The Netherlands | 6,353 | 2002.4–2006.1 | FFQ | Never  Postconception/400 | 1.00 (reference)  0.75 (0.55–1.02) |  |
| Catov 2011a | Denmark | 33,288 | 1997–2003 | interview | Never  Preconception (<1000) | 1.00 (reference)  0.84 (0.72–0.97) | age, parity, BMI, sociooccupational status, smoking, gestational age at recruitment |
| Catov 2011b | Denmark | 33,288 | 1997–2003 | interview | Never  Postconception (<1000) | 1.00 (reference)  0.92 (0.77–1.08) |  |
| Baron 2016 | The Netherlands | 2,768 | 2009.9–2011.3 | FFQ | Never  Periconception (<1000) | 1.00 (reference)  2.04 (0.91–4.55) ^*^ | age, education, ethnicity and relationship status, BMI and height, all other health behaviors/psychological characteristics, |
| Czeizel 2010 | Hungary | 13,612 | 1980–1996 | FFQ | Never  Periconception /3000 | 1.00 (reference)  1.00 (0.82–1.21) | maternal age, and socioeconomic status |

Abbreviations: **OR**, odds ratio; **CI**, confidence interval; **BMI**, body mass index; **SFFQ**, semi-quantitative food frequency questionnaire, **FFQ**, food frequency questionnaire.

^*^ OR that used the highest category of folic acid supplementation as a reference were recalculated using the lowest category as a reference to be included in the meta-analysis.
