# Supplementary material for: Folic Acid and Risk of Preterm Birth: A Meta-Analysis
Source: Front Neurosci. 2019 Nov 28;13:1284. doi: 10.3389/fnins.2019.01284 (PMC6892975; doi:10.3389/fnins.2019.01284)
Supplement: Supplementary file 5 [file Table_5.DOCX]

**Supplementary Table 5.** Characteristics of the cohort studies (n = 3) regarding the associations between dietary folate intake and the risk of preterm birth.

| **Reference** | **Country** | **Total included** | **Study years** | **Exposure analysis method** | **Folate intake (µg/day)** | **OR (95% CI)** | **Adjustment factors** |
| --- | --- | --- | --- | --- | --- | --- | --- |
| Liu 2015a | China | 10,179 | 2010–2012 | SFFQ | Preconception:  highest (median 224.6) versus lowest (median 118.6) | 0.68 (0.56–0.83) | maternal age, education level, smoking, parity, preeclampsia, maternal diabetes, preeclampsia, pre-pregnancy BMI, family monthly income per capita, maternal employment during pregnancy, history of preterm, and dietary folate intake |
| Liu 2015b | China | 10,179 | 2010–2012 | SFFQ | During pregnancy:  highest (median 272.1) versus lowest (median 155.8) | 0.57 (0.47–0.70) |  |
| Shaw 2011 | US | 5,912 | 1998.9–2005.12 | FFQ | highest (median 301.4) versus lowest (median 152.3) | 0.84 (0.64–1.08) | other two sources of folate, infant birth weight, smoking, alcohol use, race/ethnicity, maternal education, and maternal age |

Abbreviations: **OR**, odds ratio; **CI**, confidence interval; **SFFQ**, semi-quantitative food frequency questionnaire, **FFQ**, food frequency questionnaire.
