# Supplementary material for: Folic Acid and Risk of Preterm Birth: A Meta-Analysis
Source: Front Neurosci. 2019 Nov 28;13:1284. doi: 10.3389/fnins.2019.01284 (PMC6892975; doi:10.3389/fnins.2019.01284)
Supplement: Supplementary file 6 [file Table_6.DOCX]

**Supplementary Table 6.** Characteristics of the cohort studies (n = 4) regarding the associations between dietary folate intake and the risk of spontaneous preterm birth.

| **Reference** | **Country** | **Total included** | **Study years** | **Exposure analysis method** | **Folate intake (µg/day)** | **OR (95% CI)** | **Adjustment factors** |
| --- | --- | --- | --- | --- | --- | --- | --- |
| Liu 2015a | China | 10,179 | 2010–2012 | SFFQ | Preconception:  highest (median 224.6) versus lowest (median 118.6) | 0.69 (0.54–0.87) | maternal age, education level, smoking, parity, pre-pregnancy BMI, family monthly income per capita, maternal employment during pregnancy, history of preterm, folic acid supplementation |
| Liu 2015b | China | 10,179 | 2010–2012 | SFFQ | During pregnancy:  highest (median 272.1) versus lowest (median 155.8) | 0.57 (0.45–0.71) |  |
| Sengpiel 2014 | Norway | 66,014 | 2002–2009 | FFQ | none versus lowest (median 500) | 1.0 (0.61, 1.65) | maternal age, pre-pregnancy BMI, parity, history of PTB and spontaneous abortion, child’s sex, smoking habits and alcohol consumption during pregnancy, maternal education, marital status, household income, energy intake, and dietary folate intake |
| Siega-Riz 2004 | US | 3,164 | 1995–2000 | FFQ | highest (median165) versus lowest (median 950) | 1.8 (1.2–2.7) | prenatal supplement uses and batch number and batch interaction terms |

Abbreviations: **OR**, odds ratio; **CI**, confidence interval; **BMI**, body mass index; **SFFQ**, semi-quantitative food frequency questionnaire, **FFQ**, food frequency questionnaire.
